# Postmortem interval assessment by MALDI‐TOF mass spectrometry analysis in murine cadavers

**DOI:** 10.1111/jam.15210

**Published:** 2021-08-08

**Authors:** Federica Dell’Annunziata, Francesca Martora, Maria Elena Della Pepa, Veronica Folliero, Livio Luongo, Serena Bocelli, Francesca Guida, Pasquale Mascolo, Carlo Pietro Campobasso, Sabatino Maione, Gianluigi Franci, Marilena Galdiero

**Affiliations:** ^1^ Microbiology Section Department of Experimental Medicine University of Study of Campania “Luigi Vanvitelli” Napoli Italy; ^2^ Pharmacology Section Department of Experimental Medicine University of Study of Campania “Luigi Vanvitelli” Napoli Italy; ^3^ IRCSS NEUROMED Pozzilli Italy; ^4^ Legal Medicine Section Department of Experimental Medicine University of Study of Campania “Luigi Vanvitelli” Napoli Italy; ^5^ Microbiology Section Department of Medicine, Surgery and Dentistry Scuola Medica Salernitana University of Salerno Salerno Italy

**Keywords:** decomposition, epinecrotic communities, MALDI‐TOF, postmortem interval, thanatomicrobiota

## Abstract

**Aims:**

This study assessed the use of matrix‐assisted laser desorption/ionization time of flight (MALDI‐TOF) mass spectrometry as an alternative method to identify species associated with the thanatomicrobiota and epinecrotic communities.

**Methods and Results:**

The study was conducted on 10 murine cadavers, and microbiological swabs were collected from five external anatomical sites (eyes, ears, nose, mouth and rectum) and four internal organs (brain, spleen, liver, heart), during 16 and 30 days, for the thanatomicrobiota and epinecrotic communities, respectively. Our results revealed that the postmortem microbiota associated with the external cavities showed changes over time and reduced taxonomic diversity. The internal organs, initially sterile, showed signs of microbial invasion at 3 and 10 days postmortem for the liver‐spleen and heart‐brain, respectively. The postmortem microbiota was mainly dominated by Firmicutes and Proteobacteria.

**Conclusions:**

MALDI‐TOF is a promising method for estimating postmortem interval (PMI), associated with rapid sample handling, good reproducibility and high productivity.

**Significance and Impact of the Study:**

This study investigated microbial changes during the decomposition process and proposed a simple strategy for PMI estimation. Results introducing the application of the MALDI‐TOF method in the field of forensic.

## INTRODUCTION

Cadavers undergo several physical and chemical changes due to the onset of decomposition (Wescott, 2018). Decomposition is a biological multifactorial process. However, to date, the contribution of each factor is not completely understood (Singh et al., 2017). Environmental determinants, insects, body weight and clothing have significant effects on the decomposition process (Hyde et al., 2013). The different climatic conditions associated with the various geographical areas, such as arid or humid regions, play a key role in the state and rate of decomposition (Cockle & Bell, 2015). Temperature plays an important role because a warm ambient environment increases the decomposition process (Chun et al., 2015). Furthermore, temperatures ranging between 25 and 37℃ promoted bacterial proliferation (Johnson et al., 2013).

Carcass decomposition is closely related to the microbial activity (Cobaugh et al., 2015). The development of a postmortem anaerobic environment results in cell lysis and the release of biological macromolecules (Donaldson & Lamont, 2013). These substrates represent the metabolic sources of commensal microbial communities residing in the body. Bacterial fermentation cell by‐products cause swelling due to gas accumulation inside the carcass and, subsequently, liquefaction of the tissues (Hakansson & Molin, 2011). The succession of changes and decomposition products is decisive for the sequential development of microbial communities. The bacteria that colonize internal organs and microbial communities residing on the surface of decaying remains are known as thanatomicrobiota and epinecrotic communities, respectively (Dash & Das, 2020; Javan et al., 2016a, 2016b). Several studies have shown that the anatomical sites and internal organs remain sterile for up to 5 days after death (Pechal et al., 2018; Petrillo et al.,). Subsequently, bacterial migration from the gastrointestinal tract into the blood determines the bacterial spread in the spleen, heart and brain in a time‐dependent manner (Javan et al., 2019). These pieces of evidence led to the supposition that the development of the thanatomicrobiota follows regular functional and taxonomic patterns, which depend on the decomposition phase and the postmortem time.

In forensic medicine, establishing the time of death is critical (Cordeiro et al., 2019). The period between the time of death and the discovery of the body is defined as the postmortem interval (PMI) (Hurtado et al., 2018). Accurate PMI determination is important to improve our understanding of the causes of death and the dynamics of decomposition. Currently, there are several time‐dependent methods for estimating the PMI; Ceciliason et al., 2018): (i) for short periods (hours), the electrical or mechanical stimulation of skeletal muscles, and the Henssge nomogram (Gelderman et al., 2018); (ii) for intermediate times (days to weeks) the colonization of insects, fluid analysis of the decomposing body, and the chemical or biological environment characterization underlying the human remains (Dell’Annunziata et al., 2020; Iancu et al., 2018; Vass et al., 2002); (iii) for long periods (weeks to months), bone inorganic component analysis (Latham & Miller, 2019; Schwarcz et al., 2010). Naturally, the longer the PMI, the more difficult it is to precisely determine the time of death. However, these methods are subject to limitations and errors, ranging from days to months. First, body cooling ceases when the corpse reaches room temperature; as a result, the growth of insects is closely influenced by environmental factors (e.g., temperature), geographic location and season (Gelderman et al., 2018; Iancu et al., 2018; Joseph et al., 2011). Therefore, an improved understanding of new methodologies for empirically estimating PMI is mandatory. Several studies have investigated the microbiota associated with the decomposition process (Adserias‐Garriga et al., 2017). These studies evaluated postmortem microbial proliferation in the skin, internal organs, and nasal and ear cavities. Handke et al. (2017) analysed the epinecrotic communities associated with pig carcass decomposition to assess PMI. In human cadavers, changes in the bacterial composition and proliferation over time in internal organs were documented (DeBruyn & Hauther, 2017; Lutz et al., 2020; Metcalf, 2019). Tuomisto et al. (2013) examined 33 human cadavers and found that after 18 h death the blood was contaminated. However, the same authors reported that the liver and heart remained sterile for up to 5 days.

To date, knowledge regarding the anatomy and epinecrotic communities is still limited. Therefore, this study proposes the development of a simple and practical method for analysing postmortem microbiota. In murine models, epinecrotic communities from the oral, nasal, ear and ocular cavities were evaluated over a period of 30 days postmortem. Additionally, the anatomy of internal organs (i.e., liver, spleen, heart and brain) was assessed for 16 days postmortem.

## MATERIALS AND METHODS

### Animals

Ten male animals weighing 19–22 g were housed (three mice per cage) under controlled illumination (12 h light/dark cycle; light on 6:00 a.m.) and standard environmental conditions (ambient temperature 20–22℃, humidity 55%–60%) for at least 1 week before the commencement of experiments. Mice were fed chow and tap water ad libitum. The experimental procedures were approved by the Animal Ethics Committee of the University of Campania “L. Vanvitelli,” Naples. Animal care complied with Italian (D.L. 116/92) and European Commission (O.J. of E.C. L358/1 18/12/86) regulations on the protection of laboratory animals.

### Experimental design

To assess how mammalian decomposition affects microbial communities, mouse cadavers were used in laboratory settings. A total of 10 mice was divided in two experimental groups, the first for the study of the epinecrotic bacterial communities and the second group to analyse the microbiota. Murine carcasses were kept refrigerated at +4℃, under constant temperature and humidity, with insects excluded, to mimic the characteristics of the mortuary chamber. For the epinecrotic study, five mice were sampled during the initial 30 days of decomposition at regular intervals ranging from 48 h to 30 days after death (0, 2, 4, 7, 10, 14, 18, 23 and 30 days), and five external anatomical sites (eyes, ears, nose, mouth and rectum) were assessed. For the thanatomicrobiota analysis, five different mice were sacrificed, and internal organs (brain, spleen, liver and heart) were sampled at different times (0, 3, 7, 10 and 16 days). Sections of the internal organs were dissected using sterile scalpels. For each location, an individual sterile microbiological swab was rubbed while rotating the swab for a few seconds to thoroughly sample the associated microbial community. Sampling was conducted at the same time in the different stages of decomposition.

### Microbial isolation

The resulting swabs were plated on Columbia CNA agar with 5% sheep blood, MacConkey, Chocolate, Sabouraud and Schaedler agar plates (Biomerieux). All plates were incubated at 37℃ for 24 h, except for Sabouraud agar plates that were incubated at 30℃ for 48 h. Simultaneously, Chocolate and Schaedler agar plates were incubated in the presence of CO_2_ and anaerobic conditions for 48 h, respectively.

### Bacteria and yeast identification by mass spectrometry MALDI‐TOF

The cultured plates were examined, and the bacterial isolates were identified using matrix‐assisted laser desorption/ionization time of flight mass spectrometry (MALDI‐TOF) (Bruker Daltonics) (Martora et al., 2019). For MALDI‐TOF analysis, an isolated single colony from culture plates was spotted onto a 96 metallic target plate, overlaid with 1 μl of an α‐cyano‐4 hydroxycinnamic acid matrix solution and air‐dried for 5 min at room temperature. The sample matrix was introduced into a mass spectrometer for data acquisition. The sample spots were shot by laser desorption/ionization, and mass spectra, represented by mass to change ratios (*m*/*z*), were obtained. The software automatically generated the MALDI‐TOF spectrum for each micro‐organism. This spectrum instantly matched the reference database for identification. Mass spectra were obtained for each clinical isolate using Flex Control software (Bruker Daltonics). A score greater than or equal to two was attributed to species identification.

### Statistical analysis

GraphPad Prism software (version 8.0.2) was used for the statistical analysis. Different bacterial genera were compared using one‐way analysis of variance. Differences between groups were considered significant at a *p*‐value of <0.05.

## RESULTS

### Decomposition process by physical appearance

**FIGURE 1:**
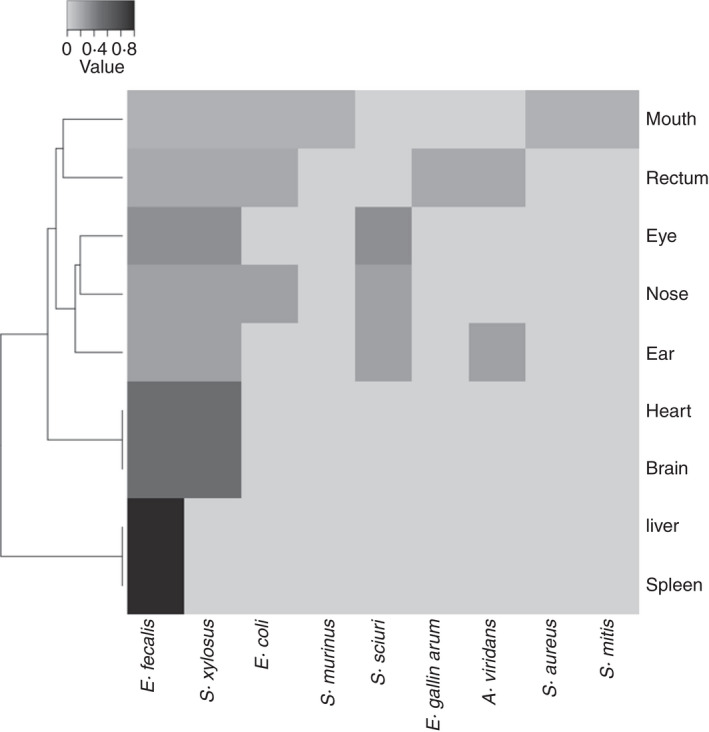
Heatmap of the relative abundances of the most predominant bacterial species associated with thanatomicrobiota and communities epinecrotic

The microbiota and epinecrotic communities were assessed over 16 and 30 days, respectively. Ten mice were sacrificed and stored at temperatures between 3 and 4℃. The decomposition phase was determined by dividing the process into five stages according to Payne's observations: fresh, swollen, early decay, advanced decay and putrid dry remains (Probst et al., 2020). All samples appeared in the fresh stage 2 days postmortem, and algor mortis and rigor mortis was observed (PMI ≥48 h). The swollen stage was verified on days 5–15. It was not discernable, possibly due to the low temperatures. During this decomposition stage, liquids were transferred from the corpses to the vessel they contained (120 h ≤ PMI ≤ 360 h). The early decay phase was evident from day 10 and progressed to advanced decay for all 30 days (240 h ≤ PMI ≤ 720 h). After 25–30 days of storage, amputation of body parts (ears, eyes, teeth, nose) was verified in some mice (putrid dry remains stage) (600 h ≤ PMI ≤ 720 h). MALDI‐TOF mass spectrometry identified the bacterial species associated with the different stages of decomposition (Figure 1). A heatmap was generated to visualize the relative abundances of the bacterial species detected in the anatomical districts for the entire period under analysis. The species were grouped in genera, and the changes over time are described as follows.

### Evaluation of epinecrotic communities

**FIGURE 2:**
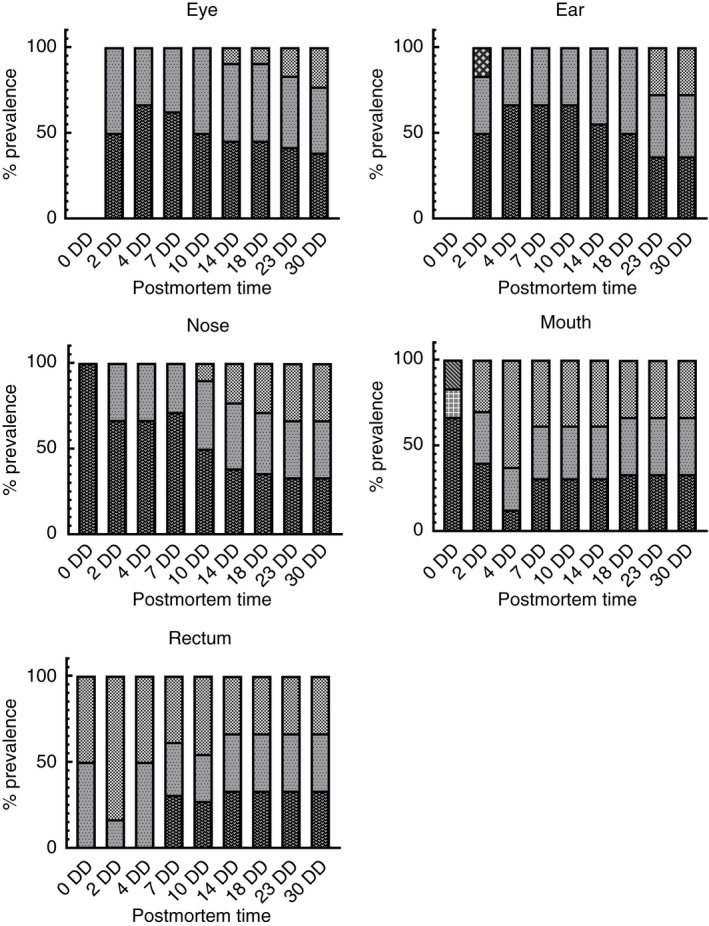
Prevalence of bacterial genera in ear (a), eye (b), nose (c), mouth (d), rectum (e) up to 30 days postmortem (*p* value <0.05) (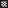 *Escherichia* spp.; 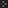 *Staphylococcus* spp.; 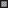 *Enterococcus* spp.; 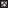 *Aerococcus* spp.; 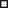 *Lactobacillus* spp.)

Changes in epinecrotic communities were monitored for 30 days postmortem. A total of 225 swabs were performed at regular intervals at five external anatomical sites (ear, eyes, nose, mouth, and rectum). In the five corpses, changes during decomposition were similar. Bacteria collected from the ear cavity showed different compositions during the postmortem period. In the initial postmortem period (time 0), no bacterial contamination was recorded. After 2 days, three different genera were registered: *Staphylococcus* spp. (50.0%), *Enterococcus* spp. (33.3% and *Aerococcus* spp. (16.7%) (PMI ≥48 h). From day 4 to day 18 postmortem, the predominant genera were *Staphylococcus* spp. (66.7%) and *Enterococcus* spp. (33.3%), and *Aeromonas* spp. were not detected (PMI ≥96 h). During the last postmortem period (from day 23 to day 30), *Staphylococcus* spp. (36.3%) were associated with *Escherichia* spp. (36.3%) and *Enterococcus* spp. (27.4%) (PMI ≥552 h) (Figure 2a). The eye sockets were sterile at time 0 postmortem. After 2 days, the right and left eyes showed the presence of bacteria belonging to the genera *Staphylococcus* spp. (50.0%) and *Enterococcus* spp. (50.0%) (PMI ≥48 h). The presence of two genera was maintained until day 10 postmortem. From day 14 to day 30, the epinecrotic community showed the presence of a new genus, *Escherichia* spp. (9.2%) associated with *Staphylococcus* spp. (45.4%) and *Enterococcus* spp. (45.4%) (PMI ≥336 h) (Figure 2b). The nasal cavity harboured bacteria of the genus *Staphylococcus* spp. at time 0 postmortem. After 2 days, *Enterococcus* spp. proliferation occurred (33.3%) (PMI ≥48 h). From day 4 to day 7, two bacterial genera were found in the nasal cavity, belonging to *Staphylococcus* spp. (71.4%) and *Enterococcus* spp. (28.6%). At 10 days postmortem, the *Escherichia* spp. was recorded (10.0%) in association with *Staphylococcus* spp. (50.0%) and *Enterococcus* spp. (40.0%) (PMI ≥240 h). Bacterial proliferation occurred until day 30 postmortem, in which *Escherichia* spp. (33.3%), *Enterococcus* spp. (33.3%) and *Staphylococcus* spp. (33.3%) were identified (Figure 2c). The bacteria collected from the oral cavity and different compositions of communities during the postmortem period were recorded. At time 0, the initial resident community included *Streptococcus* spp. (66.6%), *Staphylococcus* spp. (16.8%) and *Lactobacillus* spp. (16.7%). After 2 days, the epinecrotic communities registered changes. The genera recorded included *Staphylococcus* spp. (40.0%), *Enterococcus* spp. (30.0%) and *Escherichia* spp. (10.0%) (PMI ≥48 h). *Lactobacillus* and *Streptococcus* spp. were not detected. From day 7 to day 30 postmortem, the bacteria maintained the same composition, including *Staphylococcus* spp. (33.3%), *Enterococcus* spp. (33.3%) and spp. *Escherichia* (33.3%) on the thirtieth day (Figure 2d). The rectal cavity showed the presence of *Enterococcus* spp. (50.0%) and *Escherichia* spp. (50.0%) at time 0 postmortem. This condition reflects the commensal microbiota of the intestinal bacterial flora. After 1 week, the presence of *Staphylococcus* spp. was recorded (PMI ≥168 h). The epinecrotic community constituted by these three genera, *Enterococcus* spp. (33.3%), *Escherichia* spp. (33.3%) and *Staphylococcus* spp. (33.3%), was maintained for up to 30 days postmortem (Figure 2e).

### Thanatomicrobiota assessment

**FIGURE 3:**
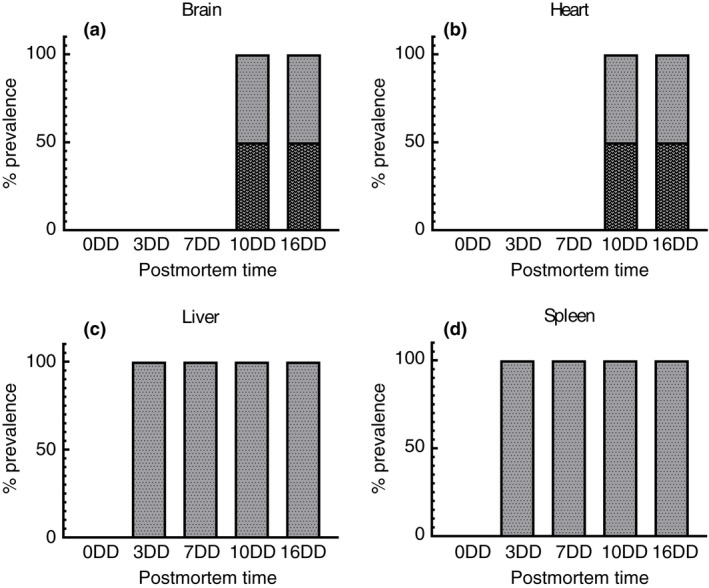
Bacterial genera assessment in brain (a), heart (b), liver (c), and spleen (d) up to 16 days postmortem (*p* value <0.05) (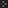 *Staphylococcus* spp.; 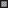 *Enterococcus* spp.)

To investigate the sequential proliferation of microbial communities in the internal organs (i.e., brain, heart, liver and spleen), five murine cadavers were dissected and swabbed at regular intervals for 16 days. Sixty swabs were tested to ensure data reproducibility. The results showed that the bacterial genera were similar between the different organs of each mouse. The brains and hearts were sterile for 7 days. Ten days after death, the bacteria belonging to *Enterococcus* spp. and *Staphylococcus* spp. genera were identified in 50%–50% of the brain and heart (PMI ≥240 h) (Figure 3a,b). The liver and spleen were sterile at 0 postmortem. After 3 days, the presence of *Enterococcus* spp. was verified, possibly due to the proximity of these organs to the intestine (PMI ≥48 h). For the entire monitored period, the *Enterococcus* genus dominated liver and spleen colonization, preventing the proliferation of other bacterial contaminants (Figure 3c,d).

## DISCUSSION

In the postmortem period, the decomposition process begins, resulting in physicochemical modifications in the corpses. Decomposition is a continuous event, and the changes that occur are important for estimating the PMI. Although the decomposition stages follow regular successional phases, it is not possible to discriminate exactly when one stage ends and the following begins. There are intrinsic factors (e.g., sex, age, regressed pathologies) and extrinsic factors (e.g., temperature, humidity and presence of insects) that influence either accelerating or slowing down the decomposition phases. Therefore, knowledge gained from other methodologies associated with physical appearance assessment is necessary.

Current studies include insect colonization and microbial community proliferation (Douglas, 2015; Iancu et al., 2018). However, these methods have limitations. The two main factors affecting insect activity are ambient temperature and body access. Furthermore, insect colonization has a different turnover depending on seasonality (DeBruyn & Hauther, 2017). In microbial communities, Javan et al. (2017) evaluated the bacterial species associated with different decomposition stages, analysed by massive parallel sequencing. This technique, although highly sensitive, requires highly qualified staff and can be costly.

Therefore, this study developed a rapid, simple and inexpensive method to determine changes in bacterial colonization over time to estimate PMI. Thanatomicrobiota and epinecrotic communities were evaluated in murine cadavers using a proteomic approach with MALDI‐TOF mass spectrometry. This methodology exhibited a very high identification capacity and cross‐validation. However, the identification procedure is based on culture‐dependent methods. This limitation is mainly related to the ability of microbes to grow under different culture conditions. Despite this limitation, our results are comparable to those obtained following the use of advanced methods. Pechal et al. (2018) conducted a follow‐up study on the decomposition process of the thanatomicrobiota in pig carcasses. The predominant phyla were Proteobacteria, Firmicutes and Bacteroides. During the decomposition process, Firmicutes and Proteobacteria showed significant changes, which are crucial for estimating PMI (Pechal et al., 2014). Moreover, Cobaugh et al. (2015) found that Proteobacteria, Firmicutes and Acidobacteria were the most represented phyla in decaying human remains. Similar changes in microbial communities were observed in this study, indicating a common decomposition pattern, which reflected the microbial composition. During the decomposition process, the epinecrotic communities associated with the eyes, ears, nose, mouth and rectum showed the prevalence of Firmicutes and Proteobacteria. In particular, the appearance of *Escherichia* spp. occurred at a precise PMI≥240, 336 and 552 h in the nose, eyes and ear, respectively. In the rectum, *Staphylococcus* proliferation was associated with a PMI of ≥168 h. In addition, the bacterial communities showed decreased diversity over time. Bacterial reduction is a parameter associated with the dominance of some phyla in response to disturbances (Zhou & Bian, 2018). In a decaying body, rapidly changing environmental conditions and the production of decay products stress some members of the community; however, others are more tolerant and proliferate. The bacterial species decrease was verified in the ear and mouth, in which at the PMI ≥48 h", the disappearance of *Lactobacillus* spp. and *Aerococcus* spp. was verified.

Furthermore, the diversity of epinecrotic communities (six phyla) was greater than that in the thanatomicrobiota (two phyla). There are two possible explanations for this finding: (i) the internal sites are typically sterile and colonized by bacterial ost‐mortem (Morris et al., 2006); and (ii) external cavities are influenced by biotic and abiotic factors rather than internal organs (Javan et al., 2016a, 2016b). In this study, all internal organs analysed were sterile up to a PMI ≥72 h. The liver and spleen were the first organs that showed Firmicutes invasion, due to the anatomical position close to the pancreas, stomach and intestine. The heart and brain were sterile during the first week. The presence of postmortem microbiota was detectable in the body at PMI ≥240 h. Therefore, the species detected were associated with the progression of putrefaction and the generalized colonization of bacteria in internal organs.

In summary, our results highlighted the bacterial dynamics of the thanatomicrobiota and epinecrotic communities as important information for estimating the time elapsed since death. MALDI‐TOF has been demonstrated as a promising method for PMI estimation. The advantages associated with the use of MALDI‐TOF are simple sample handling, short analysis time, improved reproducibility and the need for low sample quantities (Pignataro et al., 2020). Limitations are associated with the culture‐dependent method and inability to identify unculturable strains. Since MALDI‐TOF is widely used in routine microbiological laboratories, reference databases are continuously updated to increase species identification sensitivity. Implementing the identification spectrum of reference databases would increase the application of this methodology in the forensic field.

## CONFLICTS OF INTEREST

The authors declare no conflict of interest. Funding: This research received no external funding.

## AUTHOR CONTRIBUTIONS

“Conceptualization and writing—original draft preparation, Dell’Annunziata Federica and Folliero Veronica; methodology, Martora Francesca and Della Pepa Maria Elena; software, Luongo Livio; validation, Mascolo Pasquale, Bocelli Serena; formal analysis, Guida Francesca; investigation, Maione Sabatino; resources, Galdiero Marilena; data curation, Franci Gianluigi; supervision, Campobasso Carlo Pietro.
